# Compound Ammonium Glycyrrhizin Protects Hepatocytes from Injury Induced by Lipopolysaccharide/Florfenicol through a Mitochondrial Pathway

**DOI:** 10.3390/molecules23092378

**Published:** 2018-09-17

**Authors:** Wenyang Li, Ying Li, Xiangyuan Jiang, Xiaohui Li, Zugong Yu

**Affiliations:** Laboratory of Veterinary Pharmacology and Toxicology, College of Veterinary Medicine, Nanjing Agricultural University, Nanjing 210095, China; 2016107027@njau.edu.cn (W.L.); 2017107025@njau.edu.cn (Y.L.); 2017107026@njau.edu.cn (X.J.); 2017807176@njau.edu.cn (X.L.)

**Keywords:** compound ammonium glycyrrhizin, florfenicol, lipopolysaccharide, mitochondrial pathway, apoptosis, liver injury

## Abstract

Florfenicol (FFC), a widely used drug for chicken diseases, can aggravate lipopolysaccharide (LPS) damage to the liver. For this condition, natural or synthetic products displaying strong antioxidant capacity are expected to prevent LPS/FFC from inducing liver injury, so in our study, the compound ammonium glycyrrhizin (CAG) is used as the protective drug to decrease the injury to liver. The research aims to illustrate the underlying mechanism of combining LPS with FFC-induced liver injury and the protective role of CAG by using primary chicken hepatocytes as an in vitro model. The results show that LPS/FFC induced cell apoptosis and CAG protected hepatocytes from injury. The permeability of the cell membrane is elevated by LPS/FFC, leading to the efflux of enzymes (ALT, AST). Flow cytometry analysis indicates that LPS/FFC treatment increased the apoptosis rate significantly. Furthermore, with the up-regulation of apoptosis genes bax, cytochrome c and the down-regulation of bcl-2, caspase-3 and caspase-9 are activated at the gene level. LPS/FFC-induced mitochondrial damage is accompanied by a significant decrease in mitochondrial membrane potential (MMP) and severe mitochondrial damage. However, CAG improves the situation for the purpose of protecting the liver. In conclusion, it is speculated that LPS/FFC induces severe liver injury through apoptosis and the CAG protects hepatocytes from injury via the mitochondria-mediated apoptosis pathway.

## 1. Introduction

Drug-induced liver injury, the most common cause of acute liver failure in the United States [1], is a common clinical manifestation which may be a challenge to all liver related diseases [2]. Severe drug-induced liver injury causes a series of diseases which can eventually lead to hospitalization [1,3]. It is reported that antibiotics are a common cause of liver failure next to drug-induced liver injury (DILI) [4,5,6]. Florfenicol (FFC) is a broad-spectrum antibiotic for animals [7], which has great antibacterial effects on many Gram negative bacteria and positive bacteria. It has been reported that high doses of FFC aggravates liver injury with bacteriostasis [8]. Lipopolysaccharide (LPS), a major structural component of the outer membrane of Gram-negative bacteria, has the ability to cause inflammatory reactions, involving the release of a large number of inflammatory cytokines and the reduction of antioxidant enzymes and free radical scavengers [9]. If nano-LPS is mistakenly injected into the blood, it will cause septic shock in humans and symptoms of liver injury in animals [10]. The most interesting question is whether improper use of FFC in livestock production can increase the risk of liver injury at the same time of sterilization?

Although the pathogenesis of liver injuries is complex and multifactorial, the oxidative stress and mitochondrial pathway have been proposed as an important mechanism underlying the development of liver injury [11]. Therefore, antioxidants effectively protect the liver from drug-induced injury. Traditional Chinese medicine formulations such as Compound Glycyrrhizin tablets have been used for liver protection. Glycyrrhetinic acid, the main active metabolite of glycyrrhizin and glycyrrhizin, is the major water-soluble component of licorice (*Glycyrrhiza glabra*) root extract [12,13]. Both glycyrrhizin and monoammonium glycyrrhizinate have anti-inflammatory and antiviral activities, while glycyrrhizic acid is most commonly used in liver protection [14,15]. They are commonly used in the prevention and treatment of viral hepatitis. It is reported that glycyrrhizin has been shown to play an indispensable role in liver injury [16].

Although LPS and the high doses of FFC both cause liver injury, respectively, the mechanism of liver injury by LPS combined with FFC remains to be explored. Thus, the aim of the study is to reveal the mechanism of liver injury by LPS/FFC and to figure out how CAG protects hepatocytes from injury.

## 2. Results

### 2.1. The Cell Viability of Quantitative LPS Combined with Each Concentration of FFC

Firstly, to understand the effect of LPS/FFC-treated on chicken hepatocytes, MTT was used to observe the cell viability.

**Figure 1:**
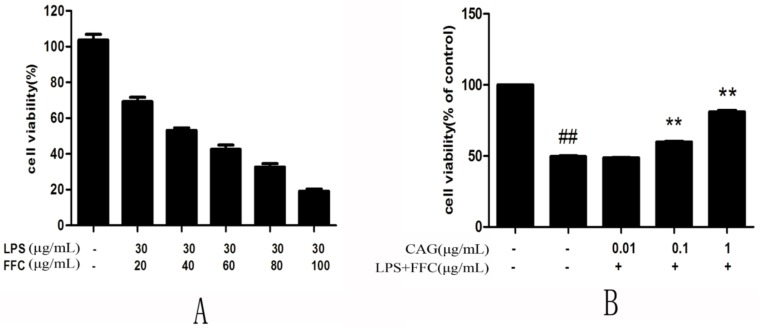

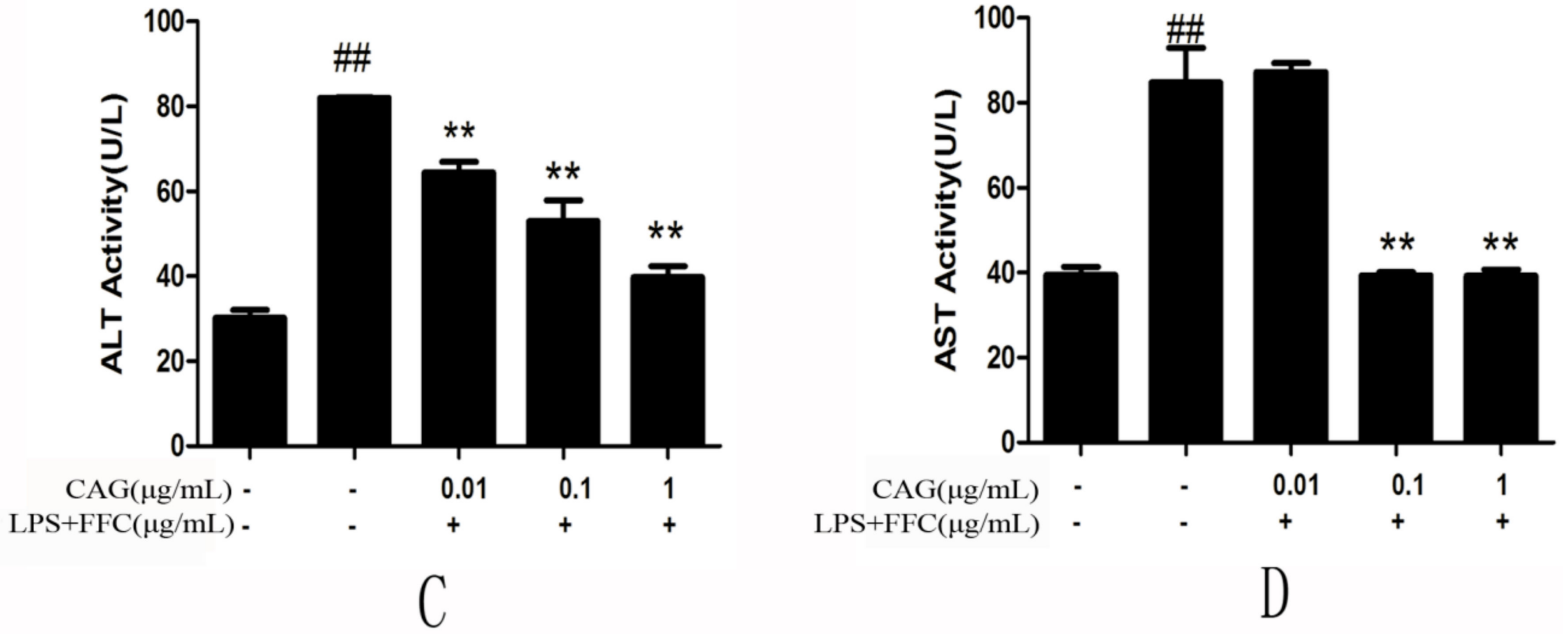
(**A**) The cell viability of hepatocytes; (**B**) The effect of CAG on cell viability; (**C**) The activity of ALT; (**D**) The activity of AST. For the LPS/FFC-treated groups. “−” and “+” mean the cells treated with normal culture medium and those treated with LPS/FFC, respectively. For the CAG groups, “−” mean cells treated without CAG. The data was expressed as the mean ± SD (n = 3). ^#^ *p* < 0.05, ^##^ *p* < 0.01 compared to the control; * *p* < 0.05, ** *p* < 0.01 compared to the model.

When treated with 30 μg/mL LPS and 20 μg/mL FFC, the viability of hepatocytes was 69.24 ± 4.32%, however, the viability of hepatocytes was 53.18 ± 2.34% at the concentration of 30 (LPS) + 40 (FFC) μg/mL. Furthermore, when the concentration was 30 μg/mL LPS and 60 μg/mL FFC, the cell viability of hepatocytes would be greatly reduced to fail the next experiment. But LPS (30 μg/mL) and FFC (40 μg/mL) caused a cell death rate of 50%, so this was considered to be the most appropriate concentration in the model group (Figure 1A).

### 2.2. CAG Alleviates LPS/FFC-Induced Acute Liver Injury in Hepatocytes

Secondly, after the concentration of LPS/FFC was determined, the hepatocytes were divided into five groups to determine the protective concentration of CAG. It showed that the viability of the hepatocytes was 49.57 ± 2.16%, about half of the control when treated with 30 μg/mL of LPS + 40 μg/mL of FFC for 24 h. However, when treated with 0.1 μg/mL and 1 μg/mL of CAG, cell viability reached 59.87 ± 1.16% and 81.01 ± 4.68% (Figure 1B). After the concentration of CAG was ascertained, the question is whether CAG can prevent hepatocytes from injury. The result indicated that both ALT and AST activities of the model group were significantly higher than those of the control group and the CAG group. The activities of ALT and AST were decreased in a concentration-dependent manner when hepatocytes were exposed to CAG. It was suggested that LPS/FFC injure the liver by destroying the hepatocytes structure and the CAG prevents hepatocytes from injury (Figure 1C,D).

### 2.3. Apoptosis Rates of the LPS/FFC and CAG Groups as Assessed by FCM

**Figure 2:**
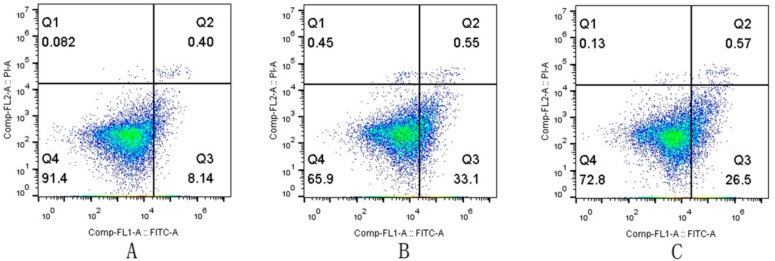

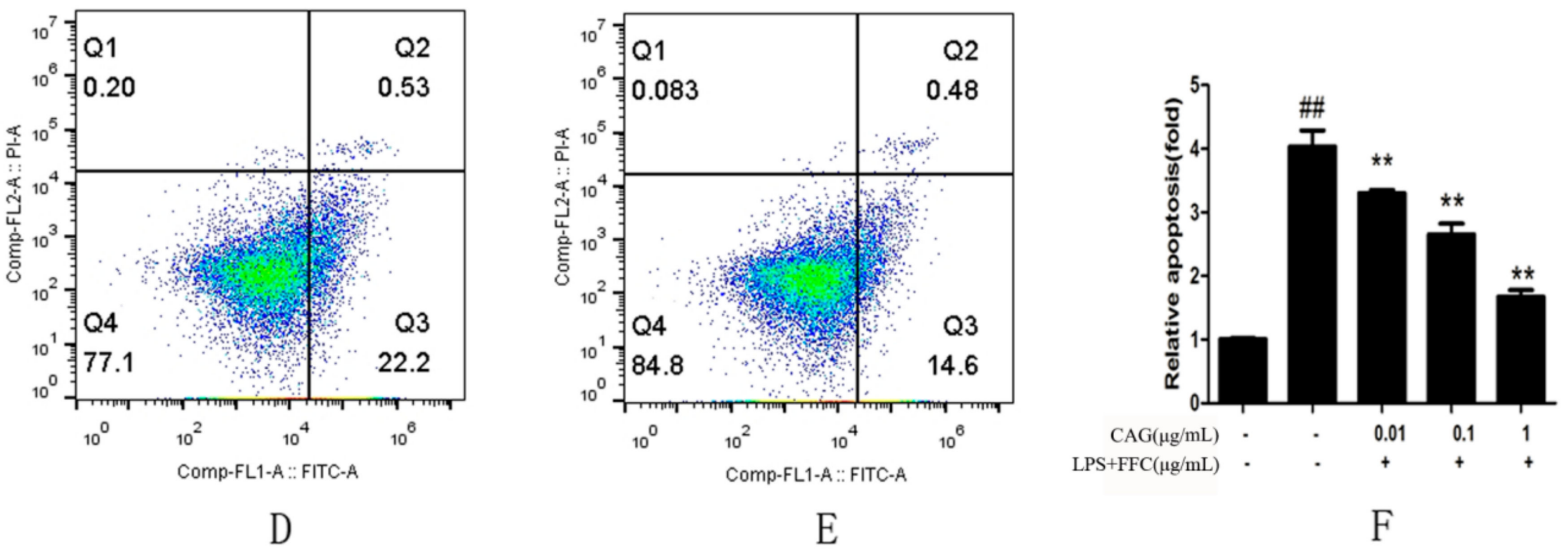
The rate of apoptosis (**A**) the control group; (**B**) the model group; (**C**) the 0.01 μg/mL CAG group; (**D**) the 0.1 μg/mL CAG group; (**E**) the 1 μg/mL CAG group; (**F**) comparison of apoptosis rates in each group. ^#^ *p* < 0.05, ^##^ *p* < 0.01 compared to the control; * *p* < 0.05, ** *p* < 0.01 compared to the model group.

Next, to further understand whether apoptosis occurred in primary chicken hepatocytes, flow cytometry was used to detect the rate of apoptosis. As shown in Figure 2, the apoptosis rate of LPS/FFC-treated hepatocytes was 33.65 ± 3.48%, the apoptosis rate of the control group was 8.54 ± 0.32% and the apoptosis rates of CAG treated hepatocytes were 27.07 ± 0.54%, 22.73 ± 2.45% and 15.08 ± 1.28%, respectively. The bar graph of apoptosis rates indicated that LPS/FFC can induce apoptosis. Moreover, the apoptosis rate decreased significantly (*p* < 0.01) when the concentration of CAG was higher than 0.01 ug/mL. It was suggested that CAG decreased LPS/FFC-induced apoptosis of chicken hepatocytes.

### 2.4. The Change of Mitochondrial Membrane Potential (MMP)

To further understand the structure of the hepatocyte injuries and whether CAG can protect hepatocytes from injury, fluorescence microscopy was used to observe the chicken hepatocytes after JC-1 staining.

**Figure 3:**
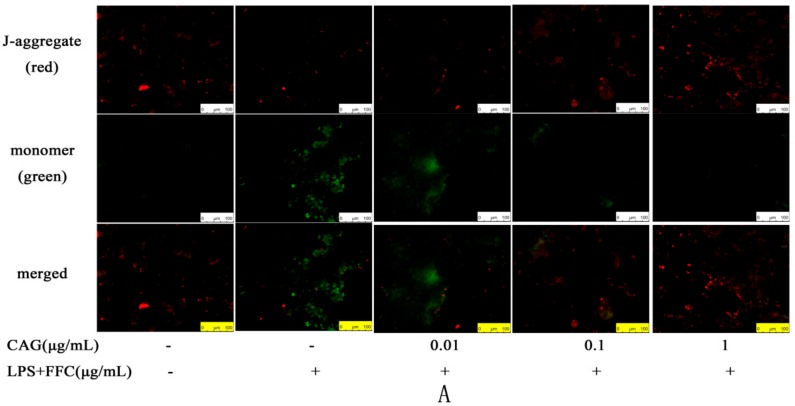

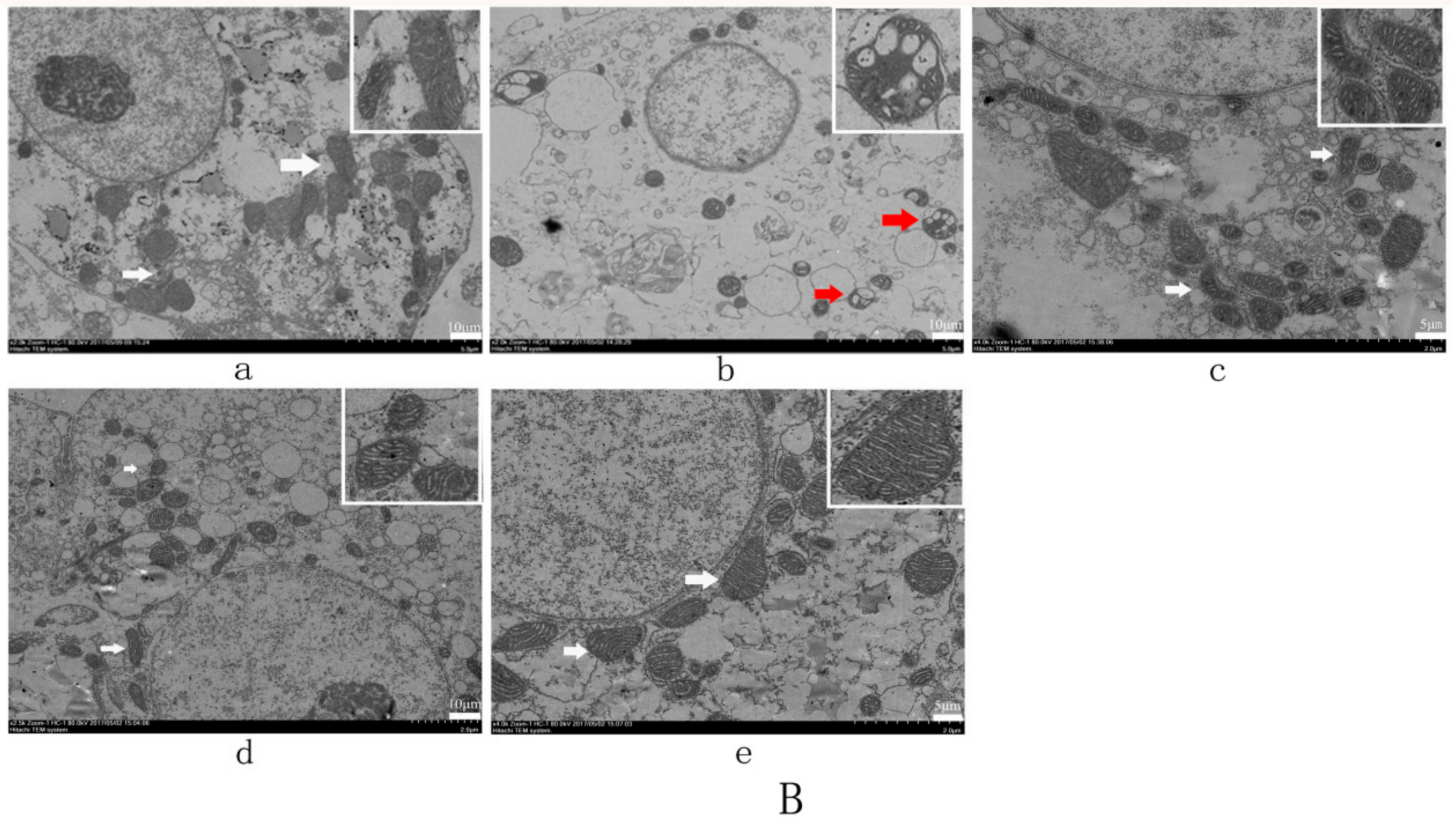
(**A**) the mitochondrial membrane potential change in chicken hepatocytes (scale bar = 100 μm). (**B**) the structural features of chicken primary hepatocytes (**a**) the control group (scale bar = 10 μm); (**b**) the model group (scale bar = 10 μm); (**c**) the 0.01 μg/mL CAG group (scale bar = 5 μm); (**d**) the 0.1 μg/mL CAG group (scale bar = 10 μm); (**e**) the 1 μg/mL CAG group (scale bar = 5 μm). The white arrow means the normal mitochondrial ridge and the red arrow means the swelling, vacuolization and degeneration of the mitochondria.

Mitochondria with normal membrane potential showed red fluorescence, while mitochondria with decreased membrane potential showed green fluorescence. Compared with the control group, the green fluorescence of LPS/FFC group was enhanced. Compared with the model group, the green fluorescence decreased with the increase of CAG concentration (Figure 3A).

### 2.5. Ultrastructural Changes in Mitochondria of Hepatocyte

After that, to further determine whether the structure of mitochondria was altered and whether CAG can protect mitochondria from damage, TEM observations were carried out. The control group showed a normal structure with normal cristae (white arrow). However, compared with the control group, the mitochondria of the model group showed swelling and vacuolization, degeneration (red arrow), while above changes were not found in the CAG group. The result indicated that mitochondrial ultrastructure was severely altered in the model group, while the CAG group protected mitochondrial from damage (Figure 3B).

### 2.6. The Change of mRNA Expression Level

**Figure 4:**
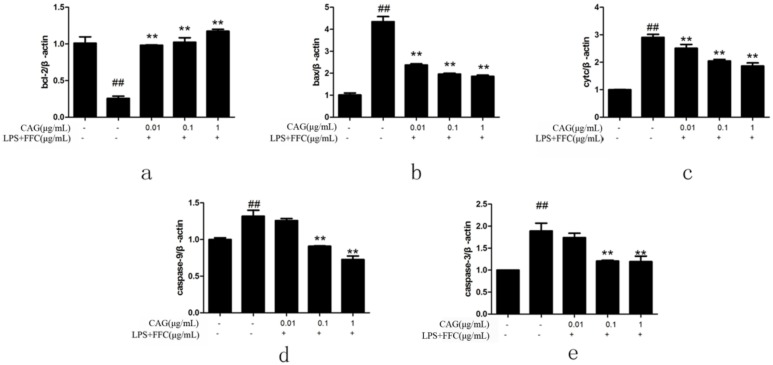
The mRNA expression of each group, β-actin was used as a reference. (**a**) bcl-2; (**b**) bax; (**c**) cytochrome c; (**d**) caspase-9; (**e**) caspase-3. The 2^−ΔΔCt^ method was used to quantify the expression levels of each gene. Values are expressed as the mean ± SD (n = 3). ^##^ *p* < 0.01 compared to the control; ** *p* < 0.01 compared to the model.

To investigate the molecular mechanism of CAG attenuated the process of LPS/FFC-induced apoptosis, RT-qPCR was used to analyze the mRNA expression level of caspase-3, caspase-9, bax, bcl-2 and cytochrome c. As shown in Figure 4, LPS/FFC significantly up-regulated the mRNA expressions of caspase-3, caspase-9, bax and cytochrome c (*p* < 0.05), while the CAG down-regulated those mRNA expressions. In addition, compared with the control group, the expression of bcl-2 in the model group was significantly down-regulated (*p* < 0.05), and compared with the model group, bcl-2 was up-regulated by CAG. That was demonstrated that CAG protected hepatocytes from damage through mitochondria-mediated apoptosis pathway.

### 2.7. Protein Levels of Cytochrome c, Bax, Bcl-2, Caspase-3 and Caspase-9 in Hepatocytes

**Figure 5:**
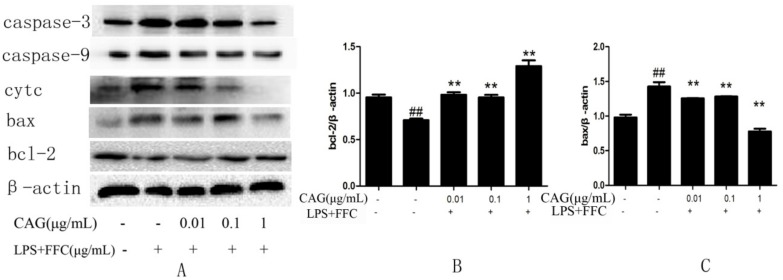

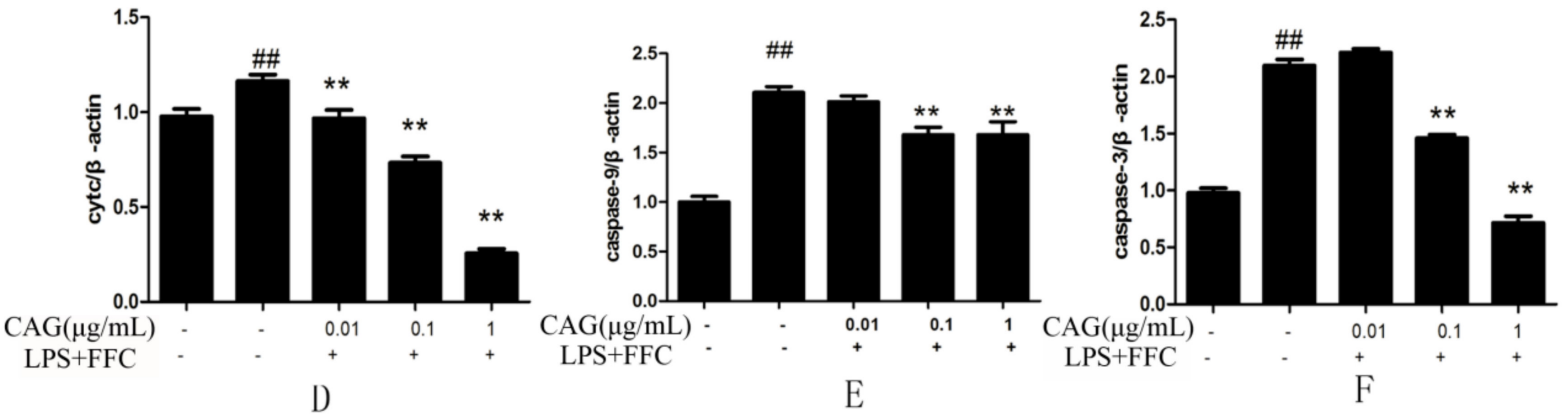
Effect of CAG on LPS/AC-induced changes in the expression of apoptosis-related proteins in hepatocytes. (**A**) western blot bands; (**B**) bcl-2; (**C**) bax; (**D**) cytc; (**E**) caspase-9; (**F**)caspase-3. β-actin is used as reference. The data are expressed as the mean ± SD (n = 3). ^#^ *p* < 0.05, ^##^ *p* < 0.01 compared to the control; * *p* < 0.05, ** *p* < 0.01 compared to the model group.

**Figure 6:**
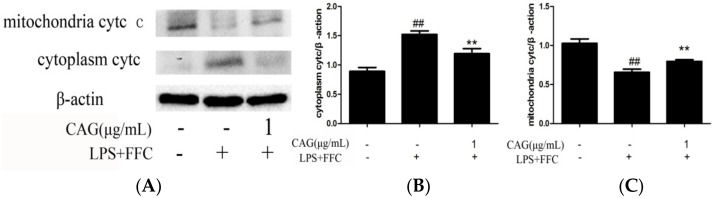
Effect of CAG on cytoplasmic and mitochondrial cytochrome c (cytc) expression in LPS/AC induced hepatocyte injury. (**A**) western bands; (**B**) cytoplasm cytc; (**C**) mitochondria cytc. β-actin was used as a reference. The data are expressed as the mean ± SD (n = 3). ^#^ *p* < 0.05, ^##^ *p* < 0.01 compared to control group; * *p* < 0.05, ** *p* < 0.01 compared to the model group.

Finally, in order to verify that LPS/FFC-induced liver damage is mediated by the mitochondrial pathway and CAG protects hepatocytes through the mitochondrial pathway, western blot assays were carried out. Compared with the control group, the protein expression levels of bax, cytochrome c, caspase-9, and caspase-3 were significantly higher in the model group. Compared with the model group, the protein expression level of the CAG group decreased as the concentration of CAG increased. However, the protein expression level of bcl-2 was significantly decreased in the model group compared to that in the control group and increased in the CAG groups compared to that in the model group (Figure 5). Moreover, Figure 6 showed that the protein expression of cytochrome c in cytoplasm was significantly higher in the model group than that in the control and CAG group and the protein expression of cytochrome c in mitochondria was significantly lower in the model group than that in the another two groups. It was suggested that LPS/FFC induced liver injury was mediated by mitochondrial pathway and CAG protected liver cells through mitochondrial pathway.

## 3. Discussion

LPS and Bacillus Calmette-Guerin or d-galactosamine has been used to establish a composite liver injury model [17,18]. Both of LPS and the high dose of FFC can induce liver injury, separately [8,18]. It was reported in different publications that glycyrrhizic acid has protective effects against experimental metabolic disorders and liver cell injury [19]. Based on the foundation, primary chicken hepatocytes were used as the in vitro model to investigate whether FFC can aggravate the liver injury caused by LPS and the protective mechanism of CAG.

In our previous studies, a modified two step collagenase IV perfusion in situ was used to isolate primary hepatocytes as a predictor of liver response [20,21]. The isolated hepatocytes showed a high level of dispersion, purity, and viability in culture for approximately nine days [22]. It enables us to have enough time to carry out a series of related experiments. On this foundation, the hepatocytes were treated with LPS/FFC that caused a concentration-dependent increase in cell death, it was indicated that the dose required for LPS combined with FFC which induced liver injury significantly lower than the LPS or FFC. It has been reported that the dose of LPS that induced primary hepatocytes injury in rats was 40 μg/mL [23]. However, the dose of LPS induced liver injury in chicken hepatocytes was higher than that in rats [22]. However, when the hepatocytes were exposed to LPS/FFC, the dose of LPS induced injury was lower than that in rats. It was speculated that FFC aggravated the liver injury caused by LPS.

In this study, the cell viability of the model group was apparently lower than that of the control and those groups treated with CAG, and the activity of ALT and AST in the cell culture medium of the model group was higher than that of the control and the groups treated with CAG. This suggested that LPS/FFC caused hepatic structural damage. ALT and AST are normally localized in the cytoplasm and ALT and AST are released into the circulation only after cellular damage [24]. However, there was no above changes in the CAG group, it was speculated that CAG can protect hepatocytes from injury. Although apoptosis in hepatocyte can occur in virus- or nonvirus-induced acute liver injury [25,26] and it is the main mechanism hallmark of liver failure [27], glycyrrhizinic acid is frequently used in these conditions for hepatic protection [15]. We, therefore, hypothesized that LPS exerted primary cytotoxicity on chicken hepatocytes by activating apoptosis, moreover, FFC can aggravate liver injury caused by LPS, however, CAG has the function of protecting against liver injury caused by LPS/FFC. In the present study, flow cytometry was performed to quantify the ratio of cell apoptosis. This suggested that LPS/FFC induced cytotoxicity was related to apoptosis induction. Furthermore, CAG restrained this effect.

The mitochondria-mediated apoptosis pathway is accompanied by mitochondrial membrane potential depolarization, and followed by pro-apoptotic molecules release from mitochondria into the cytosol [28]. The aim is to elucidate the role of mitochondria in the process of CAG attenuated LPS/FFC-induced apoptosis. The altered MMP suggested that LPS/FFC may injure hepatocytes by causing mitochondrial damage, moreover, the MMP of primary chicken hepatocytes were significantly decreased by LPS/FFC treatment and this could be reversed by CAG, suggesting that CAG may protect hepatocytes via a mitochondrial pathway. What’s more, to illustrate more intuitively whether LPS/FFC can cause mitochondrial damage and whether CAG can prevent LPS/FFC from damaging mitochondria, TEM was used to determine the ultrastructure of mitochondria. The result showed that the mitochondria appeared swollen, and vacuolated, along with blurred or even disappeared cristae in the model group, however, no such changes were observed in the CAG groups, suggesting that LPS/FFC caused severe mitochondrial damage and CAG can prevent LPS/FFC from damaging the mitochondria. Combined with the above results, it was clearly demonstrated that LPS/FFC participated in mitochondria-mediated apoptosis, while CAG can interfere with mitochondrial mediated apoptosis induced by LPS/FFC. Those data of RT-qPCR was suggested that mitochondria mediated pathway was related to LPS/FFC which induced hepatocyte apoptosis, and CAG inhibited apoptosis through the protection of mitochondria. When mitochondrial membrane permeability was changed, the decrease of MMP leads to the release of pro-apoptotic molecules into the cytoplasm and triggers downstream apoptotic processes [29]. Bcl-2 and bax are important regulators of mitochondrial mediated apoptosis, and their imbalance causes the dysfunction of mitochondria and the release of pro-apoptotic factor [30,31]. Western blot assays were used to prove it. Apoptosis is a precisely controlled programmed cell death pathway, and mitochondria-mediated apoptosis plays an important role in most of the reported pathological conditions [32]. The role of bcl-2 family proteins in apoptosis is reflected in mitochondria [33], and there are two kinds of proteins in the bcl-2 family that have the opposite effect on apoptosis: Anti- apoptotic members (for example, bcl-2) protect cells from apoptosis, whereas apoptotic members (such as bax) promote apoptosis. Oligomers formed by the interaction of bcl-2 family members transfer to mitochondria and cytochrome c is released from the mitochondria into the cytoplasm. In the cytoplasm, apoptotic bodies are formed by the interaction of cytochrome c and caspase-9, and then caspase-3 is activated. Caspase-3 degrades DNA in the nucleus, resulting in cell death. It has been reported that the proportions of bcl-2 and bax were changed in the liver tissue of fructose-fed rats [34]. Apoptosis was attenuated by CAG through regulating the bcl-2 family protein and the mitochondria-dependent cell death pathway [35]. That showed that LPS/FFC decreased the expression of bcl-2 and increased the expression of bax, cytochrome c, caspase-3 and caspase-9, while CAG increased the expression of bcl-2 and decreased the expression of bax, cytochrome c, caspase-3 and caspase-9. In combination with the mitochondrial damage induced by LPS/FFC, it was speculated that the up-regulation of bax or the down-regulation of bcl-2 family members will lead to the release of cytochrome c and further form apoptosome with caspase-9, then activate caspase-3 and induce apoptosis.

In summary, the present study highlights the importance of CAG in liver injury caused by LPS/FFC. CAG alleviates the damaging effects of LPS/FFC on hepatocytes, the release of ALT, AST and hepatocyte apoptosis. Furthermore, the mitochondria-mediated pro-apoptotic pathway is ultimately responsible for the progress of apoptosis, along with the activation of caspase-3/9, the up-regulation of bax and cytochrome c, the down regulation of bcl-2, the decline of MMP and mitochondrial damage. The good news is that CAG protects hepatocytes from injury by inhibition of the mitochondria-mediated apoptosis pathway.

## 4. Materials and Methods

### 4.1. Reagents

CAG (containing per 100 g ammonium glycyrrhizin 2.8 g, glycine and methionine 2 g each) was made in our laboratory; Florfenicol was purchased from the National Institute for the Control of Pharmaceutical and Biological Products (Beijing, China); LPS, HEPES and collagenase type IV were purchased from Sigma-Aldrich (St. Louis, MO, USA); FBS was purchased from Gibco (Los Angeles, CA, USA); Annexin V-FITC/PI apoptosis detection kit was purchased from Vazyme (Nanjing, China); Commercial kits for ALT, AST, measurement were purchased from Angle Gene Biotech (Nanjing, China). Polyclonal caspase-3 (ab115183, Abcam, Cambridge, UK), caspase-9 (ab115161, Abcam, Cambridge, UK), bax (orb334986, biorbyt, Cambridge, UK), bcl-2 (610538, BD Biosciences, San Jose, CA, USA), cytochrome c (H-104) (sc-715, SantaCruz Biotechnology, Dallas, TX, USA) and β-actin (A5441, Sigma, Saint Louis, MO, USA) were used for the western blot assays. The cells (Laboratory of Veterinary Pharmacology and Toxicology, Nanjing, China) were isolated from rooster.

### 4.2.Establishment of the Liver Injury Model

A modified two step collagenase IV perfusion in situ was used to isolate chicken hepatocytes [20,21]. A 200 μL cell solution was inoculated on 96-well plates. Cells at a density of 2 × 10^5^ were incubated for 24 h, after that, the hepatocytes were incubated with LPS/FFC concentration 30 + 20, 30 + 40, 30 + 60, 30 + 80, 30 + 100 μg/mL. Then 5 μL of MTT stock solution (5 mg/mL) were added in each well and the cells were incubated in a humidified incubator with an atmosphere of 5% CO_2_ for 4 h. The absorbance at 570 nm was measured on a microplate reader.

### 4.3. Protective Effect of CAG on the LPS/FFC-Induced Hepatocyte Injury

Hepatocytes were seeded onto 96/6-well plates for 24 h, after that, they were incubated with CAG at the concentration of 0.01, 0.1, and 1 μg/mL for 24 h. After treatment, the supernatant was discarded and the cells were exposed to LPS/FFC for 24 h. Then the cell viability was assessed as described above. The culture supernatant of each group was collected from 6-well plate. The levels of AST and ALT in the cell culture supernatant were measured by using a commercially available kit according to the manufacturer’s instructions.

### 4.4. Flow Cytometry for Apoptosis Determination

Apoptotic cells were quantified by using an Annexin V-FITC/propidium iodide (PI) detection kit and analyzed by flow cytometry. After the treatment with CAG for 24 h, the cells were treated with LPS/FFC for 24 h. The harvested cells were mixed in a 100 μL binding buffer and incubated for 10 min at room temperature with Annexin V-FITC/PI double staining solution. The apoptotic rates were measured by FAC scanning system (BD Biosciences) within 1 h.

### 4.5. Detection of Mitochondrial Membrane Potential (MMP)

Briefly, chicken hepatocytes were cultured in the previous conditions, MMP analysis was performed by using the JC-1 dye kit according to the manufacturer’s instructions. Stained cells were imaged under a fluorescent microscope at the condition of a stimulation of 490 nm and an emission of 530 nm.

### 4.6. Transmission Electron Microscopy (TEM)

After treatment with CAG and LPS/FFC, chicken hepatocytes were collected and centrifuged. The cell cluster was washed with cold phosphate buffer saline (PBS) and fastened with 2.5% glutaraldehyde at 4 °C for 1 h. After that, the samples of hepatocytes were washed twice with PBS and fastened with 1% osmium tetroxide in PBS at 4 °C for 1 h. The fastened cell clusters were dehydrated in graded series of ethanol, starting at 50% each step for 10 min, after two changes in propylene oxide. Cell clusters were embedded in araldite. Ultrathin sections were stained with Mg-uranyl acetate and led citrate for observation under an H7650 transmission electron microscope (Hitachi, Tokyo, Japan).

### 4.7. Relative Quantification of Target Gene Expression by RT-qPCR

Real-time polymerase chain reaction (RT-PCR) was used to analyze caspase-3, caspase-9, bax, bcl-2 and cytochrome-c. After treatment with LPS/FFC and CAG, TRIzolTM (9108/9109 Takara, Tokyo, Japan) was used to extract total RNA and gDNA eraser was used to wipe off any potential genomic DNA. The purity and concentration of RNA were determined by Nano Drop 2000 (BIO-RAD, Philadelphia, PA, USA). PrimeScript™RT reagent kit (RR047A Takara) was used for cDNA synthesis. Moreover, SYBR green premix (RR820A Takara) was used for RT-PCR analysis. The PCR program includes the cycle of 95 °C for 30 s, 95 °C for 5 s and 60 °C for 30 s 40 times. The relative quantity of the target gene mRNA = 2^−△△Ct^, where ΔΔCt = (Ct_Target gene_ − Ct_β-actin_)test group − (Ct_Target gene_ − Ct_β-actin_)control group.

### 4.8. Western Blotting

Hepatocytes were homogenized in RIPA buffer solution, which consists of 50 mM Tris/HCl (pH = 8), 150 mM NaCl, 1%non-idet-P40, 1%sodium deoxycholate, 0.1% SDS, 0.1 mM DTT, 0.05 mM PMSF, 0.002 mg/mL aprotinin, 0.002 mg/mL leupeptin and 1 mM NaVO_3_. The concentration of the upper protein was determined by the BCA kit following the manufacturer’s instructions. The equal number of protein samples were electrophoresed on SDS–PAGE (12% polyacrylamide gels) and transferred to nitrocellulose membranes (Immobilon, Millipore Corp, Bedford, MA, USA), then the nitrocellulose membrane was incubated with antibody. After incubation, TBST was used to wash the membrane and then ECL reagent was used to detect the immune reaction band. Western blot bands were detected by Quantity One software (Bio-Rad, Hercules, CA, USA). The data was normalized to the corresponding β-actin (A5441, Sigma, Saint Louis, MO, USA), which served as a reference.

### 4.9. Statistical Analysis

Statistical analysis was performed by using SPSS 19.0 (IBM Corp., Armonk, NY, USA) and GraphPad Prism 4 (GraphPAD Software, San Diego, CA, USA). All data were presented as mean ± standard deviation. One-way analysis of variance (ANOVA) followed by Tukey’s test was used for multiple comparisons, and *p* < 0.05 was considered as significant compared with different groups.
